# Comparative analysis of the impact of portable digital X-ray on TB screening in hard-to-reach areas in Nigeria

**DOI:** 10.5588/pha.24.0040

**Published:** 2025-03-01

**Authors:** S. Useni, B. Odume, M. Tukur, E. Chukwu, N. Nwokoye, C. Ogbudebe, O. Chukwuogo, U. Osuoji, C. Igwetu, I. Gordon, D. Nongo, R. Eneogu, A. Ihesie, O. Chukwuekezie, C. Anyaike, M.Y. Diul

**Affiliations:** ^1^ KNCV Nigeria, Programmes, Abuja, Nigeria;; ^2^ USAID Mission Nigeria, Abuja, Nigeria;; ^3^ Department of Community Medicine, University of Nigeria Teaching Hospital, Enugu, Enugu State, Nigeria;; ^4^ Federal Ministry of Health, Public Health, National Tuberculosis, Leprosy and Buruli Ulcer Control Programme, Abuja, Nigeria;; ^5^ USAID, Infectious Disease Office, Tuberculosis Division, Global Health Bureau, Washington, DC, USA.

**Keywords:** PDX, computer-aided diagnosis, CAD, CAD4TB, chest X-ray

## Abstract

**SETTING:**

TB screening cascade and performance of active case-finding strategies across six states of Nigeria.

**OBJECTIVE:**

To analyse the impact of portable digital X-ray (PDX) on TB screening in hard-to-reach areas in Nigeria.

**DESIGN:**

A cross-sectional study involving enrollees with a CAD4TB score of ≥50 had Xpert (sputum) and/or radiographic assessment for TB diagnosis. A TB screening algorithm guided the step-by-step process of identifying a presumptive TB client up to diagnosis and linkage for appropriate treatment. Data were collected, collated, and reported using the national TB tools.

**RESULTS:**

Seven PDX with CAD4TB used as TB screening and diagnostic tools across six states screened 85,910 persons and identified 8,416 presumptive TB cases. From this study, PDX had the lowest number needed to screen (NNS) of 45 and the number needed to test (NNT) of 4. Similarly, PDX, with a presumptive TB yield of 10%, had the highest TB yield of 23%.

**CONCLUSION:**

Using PDX with CAD contributed to the highest TB yield during Active TB case finding in hard-to-communities of Nigeria. With a very low NNS and NNT, its national scale-up and use across remote locations will significantly improve TB case-finding.

Nigeria is among the 10 countries with the highest burden of TB, TB-HIV and drug-resistant TB (DR-TB). With an incidence rate of 219/100,000 and an estimated absolute number of 479,000, Nigeria is ranked first in Africa and sixth globally. Although Nigeria accounts for about 2.8% of the world population, it accounts for 4.6% of the global TB burden.^1,2^

There are millions of people not diagnosed with TB that are infecting and re-infecting other people. These missed cases are widening the gap between the estimated number of people who fell ill with TB and the number newly diagnosed.^2,3^ The number of people dying of TB in 2022 as a result of TB was 156,000. This figure included 28,000 who lived with HIV.^3,4^

The problems mitigating against any progress in the fight against TB in Nigeria are many and evolving. There are obvious effects of HIV and the multidrug-resistant TB (MDR-TB).^3–6^ There are issues of seemingly unending insecurity in the forms of terrorism, banditry, migration and kidnapping.^4–6^ Other issues include gaps in the government’s stewardship role, along with the associated challenges in coordinating certain aspects of TB control services. There are sometimes irregular supplies of drugs, as well as recording and reporting tools to the facilities and the patients.^3,4^ The human resources are poorly paid, poorly motivated and poorly deployed. The Technical Partners are seemingly fatigued due to inadequate funding and sometimes an absence of the supportive role of the government.^4^

There are other challenges like untoward terrain, seasonal emergencies such as erosion, landslides, wildfire, storms with wind and the difficult vegetation in the communities in Nigeria.^5,6^ These problems are collectively or singly linked to low case-finding, poor contact tracing, unimpressive case-holding, and the increasing number of TB cases, including MDR-TB cases in the country.^4,5^

During the 37^th^ Stop TB Partnership Board Meeting in 2023, the Federal Ministry of Health (FMoH), Nigeria, proposed a promising approach in Brasilia, DF, Brazil. Nigeria diagnosed over 300,000 TB cases for the first time since the inception of the National Tuberculosis and Buruli Ulcer Control Program (NTBLCP).^3,4^ This feat was achieved with the Technical Partners supporting the national TB control services at various Federal, State and Local Government tiers. KNCV Nigeria is one of the partners.^7^

With funding from the USAID TB local organisation network project, KNCV Nigeria re-invigorated the public-private mix, articulated massive advocacy and social mobilisation, and cascaded training and re-training of frontline health workers and volunteers at the subnational levels across 14 states of Nigeria.^7–9^

Similarly, a collaboration between the Stop TB Partnership and the United States Agency for International Development ushered in the Introducing New Tools Project (iNTP).^8,10^ These new tools support diagnostics, treatment, and digital health technologies and mainly facilitate the diagnosis of missed and underdiagnosed cases of TB in high-burden countries.^8,10,11^

KNCV Nigeria, in line with the efforts of Stop TB Partnership and USAID, deployed the portable digital X-ray with computer-aided diagnosis (PDX with CAD) received as TB screening and/or diagnostic tool for hard-to-reach communities in Benue, Cross River, Delta, Kano, Katsina, and Nasarawa States in Nigeria.^7–9^

The PDX with CAD operates based on the same principle as the traditional X-ray devices.^11^ It is portable battery-operated equipment and has better storage and retrieval. It is easy to use, versatile, and efficient, with possible electronic transmission of images, appreciably good quality images and a good shield that helps to protect both the patients and the users.^11^

CAD has some limitations like long-term high cost due to maintenance issues over time resulting from tear and wear of components, not indicating the absence and presence of diseases like HIV. There could be confusion in scarring due to TB. PDX with CAD allows for the screening of TB in remote and hard-to-reach areas without health, electricity, or communication facilities.^7,9,10^

The CAD4TB (Delft Imaging, Delft, The Netherlands) software (computer-aided detection for tuberculosis) provides a tuberculosis probability score ranging from zero (0) (low probability) to 100 (high probability).^12^ The score indicates the likelihood of TB presence in the person shown in the image.

There are different versions of the CAD4TB with different performances.^12–14^ It was found that CAD4TB v4 and CAD4TB v5 outperformed human readers (85.9% and 87.0% versus 85.2% for human readers.^13,14^ Through this new tool project, the PDX machines by Delft Imaging use CAD4TB v7.^13,14^ Data are available measuring the performance of CAD4TB in children aged ≥4 years. In the field, the CAD4TB is used both online and offline, and the CAD data are stored in the cloud.^14^ There are operational threshold scores given by the manufacturers for different versions of CAD4TB.^13,14^

This paper aims to analyse the impact of PDX with CAD on TB Screening in hard-to-reach areas in Nigeria.

## DESIGN AND METHODS

In January 2022, KNCV Nigeria deployed seven PDX CAD4TB scores ≥50 as a threshold guide for patients to be sent for Xpert^®^ MTB/RIF (Cepheid, Sunnyvale, CA, USA) and/or clinical (radiograph) assessment for TB diagnosis. There was an initial engagement of the leadership of the NTBLCP and other key stakeholders, and a road map was developed.

The national TB guidelines and training materials were adopted, and nationwide awareness and sensitisation were done. In collaboration with the state TB programs, high TB burden communities and target populations were identified using hotspot analytics in the form of an early warning outbreaks recognition system (EWORS), followed by advocacy visits to the major stakeholders. Before equipment installation at the selected facilities and deployment for use in the communities, hands-on staff training was conducted, followed by periodic mentorship and supportive supervisory visits.

This study is a cross-sectional study of retrospective data from the 7 PDX machines with CAD as a TB screening and diagnostic tool for hard-to-reach communities across six states of Benue, Cross River, Delta, Kano, Katsina and Nasarawa.

Cascade data was collected to demonstrate the impact of TB screening on enrollment. Data compared TB screening cascade for ACF using PDX with other supported ACF interventions without PDX: intensified case finding (ICF) in public facilities, ICF in private facilities, patent and propriety medicine vendors (PPMVs), contact investigation (CI) and community pharmacy (CP) in the same states. A comparison was also made between the PDX and other interventions in terms of NNS and NNT to measure efficiency.

Targeted populations include internally displaced persons (IDP) camps, markets, health facilities, mobile populations, prisons and other relatively hard-to-reach groups and areas without mobile networks and internet connectivity. TB screening was conducted, and the activity spanned 5 days in a week under strict standard safety precautions for all eligible age groups, excluding pregnant women and children aged <6 years. The NTBLCP reporting and recording tools were used to collect and collate the data.

A TB screening algorithm (Figure) guided the step-by-step process of identifying a presumptive TB client up to diagnosis and linkage for appropriate care and treatment.

**FIGURE.**
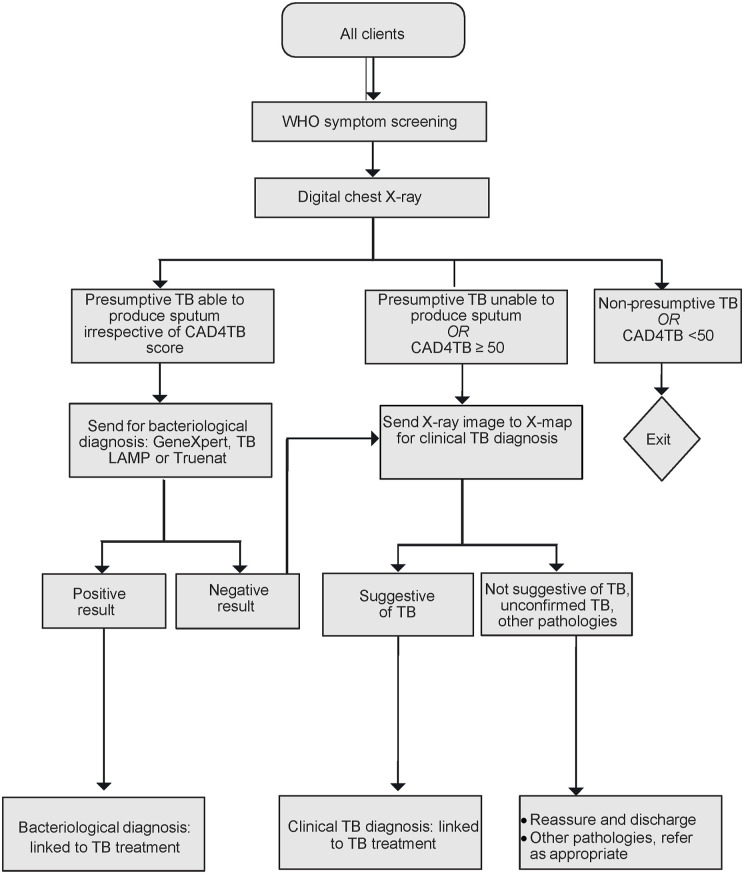
Flow diagram of the community (including hard-to-reach areas, groups and those at high-risk) TB case-finding interventions (modified from Babayi et al.^20^). Parallel TB screening algorithm with DLB and WoW truck digital X-ray. DLB = Delft Light Back; WoW = Wellness on Wheels; TB LAMP = tuberculosis loop-mediated isothermal amplification.

### Ethical considerations

As this study did not involve human subjects, ethical clearance was not required. The data utilised in this study were collected as part of routine project implementation and did not include any personal or sensitive information. All procedures adhered to standard ethical guidelines for data collection and handling, ensuring confidentiality and privacy

## RESULTS

### Portable digital X-ray

Screened 85,910 persons and identified 8,416 presumptive TB cases. Among these, 1,915 TB cases were diagnosed, and 1,869 patients were put on treatment. The number needed to screen (NNS) was 44.9, indicating that for every 44.9 people screened, one TB case was diagnosed.

### Intensified case-finding in public facilities

A total of 209,975 presumptive TB cases were identified, resulting in 16,559 diagnosed TB cases, of which 16,124 patients received treatment. The NNS was 205.6.

### Private facilities

A total of 462,120 individuals were screened, identifying 31,305 presumptive TB cases, diagnosing 2,581 TB cases, and providing treatment to 2,501 patients. The NNS was 179.0.

### Patent and propriety medicine vendors and community pharmacists

A total of 1,481,066 individuals were screened, leading to the identification of 169,498 presumptive TB cases, the diagnosis of 11,589 TB cases, and the treatment of 11,443 patients. The NNS was 127.8.

### Contact investigation

A total of 160,828 individuals were screened, resulting in the identification of 48,469 presumptive TB cases, 3,282 diagnosed TB cases, and the treatment of 3,146 patients. The NNS was 49.0.

### Community active case-finding

Screened 1,298,202 persons, identified 93,460 presumptive TB cases, diagnosed 3,342 TB cases, and treated 3,273 patients. The NNS was 388.5 (Table).

**TABLE.** Cascade of TB case-finding strategies across KNCV Nigeria facilities, 2022.

TB case-finding strategy in 2022	Persons screened	Presumptive TB identified	Presumptive TB evaluated for TB	TB cases diagnosed	TB patients put on treatment	NNS	NNT
PDX	85,910	8,416	8,401	1,915	1,869	44.9	4.5
ICF public facility	3,404,939	209,975	196,406	16,559	16,124	205.6	11.9
ICF private facility	462,120	31,305	31,086	2,581	2,501	179.0	12.0
PPMV/CP	1,481,066	169,498	160,758	11,589	11,443	127.8	13.9
Contact investigation	160,828	48,469	45,890	3,282	3,146	49.0	14.0
Contact investigation: public	124,564	35,772	33,399	2,396	2,282	52.0	13.9
Contact investigation: private	36,264	12,697	12,491	886	864	40.9	14.1
Community ACF	1,298,202	93,460	84,974	3,342	3,273	388.5	25.4

NNS = number needed to screen; NNT = number needed to test; PDX = portable digital X-ray; ICF = intensified case-finding; PPMV = patent proprietary medicine vendor; CP = community pharmacy; ACF = active case-finding.

## DISCUSSION

In 2022, 85,910 people were screened for TB, and 8,416 presumptive TB cases were identified using PDX with CAD intervention for TB screening diagnosis, treatment, and notification in hard-to-reach communities across six states of Benue, Cross River, Delta, Kano, Katsina and Nasarawa states. Among these, 1,915 TB cases were diagnosed, and 1,869 (98%) patients were placed in treatment. The NNS was 44.9, indicating that one TB case was diagnosed for every 45 persons screened. The use of PDX accounted for approximately 50% of all clinically diagnosed TB patients.

During the same period, 3,867,059 persons were screened for TB in public and private health facilities, 19,140 TB cases were detected, and 18,625 (97.3%) were successfully placed on treatment. The NNS was 205.6 and 127.8, respectively. The NNS was quite high compared to 44.9 in the PDX-CAD system.

Also, 160,828 contacts of index TB cases were traced, and 48,469 persons were identified as presumptive TB cases. Of that number, 45,390 presumptive TB cases were evaluated for TB, 3,282 TB cases were diagnosed, and 3,146 (95.8%) patients were put on treatment. The number diagnosed was high but comparatively lower than the PDX-CAD system.

During community outreaches for TB case finding, the PDX-CAD intervention screened 1,298,202 people. The presumptive TB cases identified were 93,460, and those evaluated were 84,974, of which 3,342 TB cases were diagnosed and 3,273 (97.9%) were enrolled for treatment. The NNS was 388.5. Hot spots were identified for these targeted community outreaches, and TB screening was conducted in those areas. TB cases diagnosed are promptly placed on treatment.^6,15–21^ It was observed that PDX-CAD was the more efficient intervention.

The engagement of Patent and Propriety Medicine Vendors (PPMVs) and Community Pharmacists is also a creative way of finding the missing cases of TB in the communities and hard-to-reach areas and groups. They are close, cheaper, trusted and preferred by many people.^22–24^ Through this intervention, 1,481,066 persons were screened for TB, with 169,498 presumptive TB cases identified and 160,758 evaluated. The TB cases diagnosed were 11, 589, and 11,443 (99%), and they were placed on treatment. The NNS was 127.8, and nearly all the patients were put on treatment because they were known and could be traced to their residence.^19,23,25^ However, the PDX-CAD system presented a better intervention.^21,23^ This is in line with the findings by Arinze et al.^23^ and Daftary et al.^20,23^ Odume et al.^16^ in earlier studies observed that TB screening using the PDX during active case-finding in hard-to-reach communities in Niger Delta, Nigeria, showed a high prevalence of TB among those screened. The study pointed out the need for nationwide use of PDX. The use of the PDX-CAD system in hard-to-reach areas and high-risk populations has made healthcare accessible and acceptable to many who would have missed the opportunity.^6,7,16,17^

The evaluation of CAD4TB on chest radiography among people living with diabetes in Pakistan showed that CAD4TB offers good diagnostic accuracy as a triage test for TB screening among people with diabetes.^25^ Xpert was used as the reference standard.^21,25^ Three (including CAD4TB v7) CAD software solutions performed at par with the expert, and three additional CAD software solutions performed at least on par with the intermediate reader.^25^

The newer version of CAD4TB software was observed to outperform all previous versions by achieving 98% specificity at 90% sensitivity.^26^ In a study by Fehr et al. in rural South Africa, the team observed that CAD4TB v5 performed like the radiologist in triaging participants for diagnostic sputum testing. The study further elucidated that using CAD4TBv6 to triage digital CXRs will increase the yield of active case-finding activities compared with symptom-based triage, which is feasible in rural settings. Different versions of the CAD4TB have different efficacies.^26^ Codlin, in evaluating the performance of newer versions of CAD4TB, demonstrated reduced effectiveness in individuals with a history of TB.^27^ Babayi et al.,^20^ in an African setting, reviewed the efficiencies of the interventions adopted to improve TB case-finding. The PDX was outstanding compared to other interventions like intensified case finding in private and public facilities, targeted community outreaches, active case finding in nomads, contact investigation and wellness on wheels.

## CONCLUSION

Many interventions have been adopted to improve TB diagnosis and notification in Nigeria’s TB control programme. The use of the PDX-CAD system, compared to other active TB case-finding interventions, significantly improved the number and efficiency of TB cases diagnosed and put on treatment. There is a need for continuous advocacy by policymakers, technical partners, and other stakeholders to increase funding and support for procuring and deploying newer TB screening tools, including PDX with CAD. The National Programme should support regular maintenance of the PDX-CAD system, periodic supervision and hands-on mentorship of the staff involved in the use of PDX with the CAD system.
